# Catharsis Through Cinema: An Italian Qualitative Study on Watching Tragedies to Mitigate the Fear of COVID-19

**DOI:** 10.3389/fpsyt.2021.622174

**Published:** 2021-06-16

**Authors:** Ines Testoni, Emil Rossi, Sara Pompele, Ilaria Malaguti, Hod Orkibi

**Affiliations:** ^1^Department of Philosophy, Sociology, Education and Applied Psychology (FISPPA), University of Padova, Padua, Italy; ^2^Emili Sagol Creative Arts Therapies Research Center, University of Haifa, Haifa, Israel

**Keywords:** COVID-19, cinema, qualitative research, epidemic, death, catharsis

## Abstract

**Background:** Among different ways of coping with the unsettling situation of the COVID-19 pandemic, a very peculiar one has been identified: a more frequent request, by the general population, of movies or TV series related to the very theme of viruses, contagions, and epidemics.

**Objectives:** The aim of the present study was to explore this peculiar phenomenon, in order to identify people's emotions and cognitions during and after the process, and to better understand the possible psychological function cinema can have during moments of intense and generalized crisis like the present COVID-19 pandemic.

**Participants:** Fifteen Italian adults took part in the study – eight women and seven men (average age = 30 years, SD = 10.54). Participants were recruited through social media via a specific announcement, and subsequently, through a “snowball sampling.”

**Method:** For the present study a qualitative approach was adopted, and more specifically, the principles of Interpretative Phenomenological Analysis (IPA) have been followed. Semi-structured interviews were conducted by telephone or through online meeting platforms (Zoom or Skype). The written texts obtained from the transcription of each interview were analyzed using thematic analysis with the support of the software *Atlas.ti*, in order to highlight their fundamental contents.

**Results:** From the data analysis, four main areas of thematic prevalence emerged, which reflected the participants' condition during the pandemic that actually led them to watch epidemic-themed movies or TV series: the need to document themselves concerning the theme of epidemics, the need to exorcize contagion anxiety, the desire to find a character with which they could identify, and the casual condition of finding a peculiar movie related to the theme of pandemics and being interested in it because of the striking resemblance with real life.

**Conclusions:** The findings suggest that cinema could indeed represent a powerful tool to help people face the intense uncertainty of the new COVID-19 epidemic, since it allows spectators to both obtain more insight into the current situation, and to project their fears and uncertainties into the movie or TV series, thus reaching a sort of cathartic liberation that offers them hope toward the future.

## Introduction

In 2020 humanity had to face the new threat of a pandemic. A new type of Coronavirus, the COVID-19, spreading rapidly from China has affected the entire world population, with a devastating impact on both the public health and the global economy (1, 2). For these reasons, the World Health Organization (WHO) on the 31st of January 2020 declared the state of public emergency of international interest, and on the 3rd of March confirmed the presence of a global pandemic (3).

Italy has been the first Western Country that had to face firsthand the consequences of the epidemic. The first cases of COVID-19 were registered on the 21st of February (4), with a total amount of recorded cases until the present day of more than 434,449 (5). Since the first cases in February, the spread of the contagion had grown rapidly to few hundreds of ill people in a few days, so that the Italian government decided to create the first so called “red-zones,” that is, special secluded areas corresponding to the towns and cities most affected by the number of contagions. Inside red-zones, people were forbidden from leaving their homes except for essential needs (groceries shopping, sanitary reasons, urgent work reasons or other serious emergencies) (6). However, as neither this measure proved to be enough to stop the spread of the virus, the Italian government decided in March 2020 to enact a total lockdown on the whole national territory that would endure for two months (from the 8th March to the 3rd May 2020).

Many studies that have been conducted in the immediate period following the lockdown, both in Italy and in the other countries equally affected by the pandemic, have highlighted how the situation of health emergency and the social isolation imposed during the lockdown seriously impacted on the general population, also in terms of mental health and general well-being (7). A recent systematic review conducted by Hossain et al. (8) has pointed out how the imposed quarantine and the isolation measures negatively affected mental health. This trend has also been confirmed by other studies (9–11) describing a serious increase in the rates of anxiety, panic attacks, obsessive–compulsive disorder, stress, and trauma-related disorders in the population during and right after the lockdown (12, 13), even when compared to the already serious effects of the 2020 SARS epidemic per se (14).

The COVID-19 epidemic has in fact spread much more than any previous one, leading to a global condition of fear and uncertainty that is still highly present. People from all over the world have been therefore forced for many months to confront themselves with their worst and best-hidden fear: death (15). This highly distressful situation has grown so significantly that researchers have coined the term “pandemic stress” as a typical stress syndrome of 2020 (16).

Especially for Western society the theme of death and dying is still too frequently hidden and concealed and the thought of human finitude is suffocated, and this because of a substantial inability to manage the extreme anguish that death generates (17). This mechanism of repression has been explored in depth by Terror Management Theory (TMT) (18). TMT suggests that this common worldwide fear of death actually guides the majority of people's choices in everyday life, from the pursuit of social self-esteem to the religious affiliations and even in political preferences. According to the TMT, the thought of death is usually counteracted by the so called “anxiety buffers” that either suppress and/or project it in a far and undetermined future, allowing people to feel invulnerable again (18).

COVID-19 has now challenged this typical balance, leading to a striking and constant mortality salience (15). Since very little was known about this new threat and both governments and media seemed hesitant and contradictory in giving information, a paralyzing uncertainty and fear took citizens (19).

Particularly during the lockdown, the anguish of not knowing anything about the new danger had become extremely intense, leading people to adopt certain kinds of behaviors that could result very peculiar and difficult to explain. One of those was deciding to watch movies or TV series directly related to the themes of contagion, epidemic, pandemic etc., exactly during the period of the highest peak of COVID-19 contagions (20, 21). Such a behavior seems to disprove TMT itself, according to which, as it has been mentioned before, people would generally feel the need to suppress any thought that could be linked with death and/or human vulnerability (22–24).

However, this phenomenon had happened before. Some researchers reflected on the fact that there are similarities between the themes represented in a movie that pertains to this peculiar thematic category and how viewers perceive what can actually happen during a real pandemic (25), sometimes even with some counterproductive outcomes, especially when the representations in such movies are not adequately realistic and cannot therefore offer proper guidance concerning what to do and how to behave (26). Nevertheless, it has been suggested that a movie, as well as a TV series, can potentially become a dimension that leads people to stop and reflect upon this matter, sometimes even to a strategy to interpret reality (27). Giving images of either realistic or completely fictitious worlds that constitute a mirror of the spectators' present reality can help them focus on certain essential traits and aspects of life itself, being this particularly true for movies concerning epidemics (28).

This process was explored in depth by French philosopher and sociologist Edgar Morin (29). He would describe cinema as an “anthropologic mirror” of human nature, capable to generate in the spectator a perception that moves in a sort of double-conscience: an illusory one (of identification with the story narrated in the movie) and a real one (the part of the spectator that remains anchored to his/her actual everyday life) (29). In this way, cinema can offer people the possibility to enter a new world, without however feeling too disoriented, in a kind of aesthetic transfiguration that also allows people to discover the real world itself. According to the author, the emotional intensity with which the spectator watches a movie triggers a sort of cognitive metamorphosis (29).

It is precisely inside this thematic frame that the present study is inserted. We sought to further explore the peculiar phenomenon of people who, precisely during a devastating pandemic and a period of strict lockdown, felt the desire to watch movies or TV series linked with the theme of viruses, contagions and epidemics, in order to identify their emotions and cognitions during and after the process. It was also our aim to better understand the possible psychological function cinema can have during moments of intense and generalized crisis like the present COVID-19 pandemic.

## Materials and Methods

Fifteen Italian people took part in the study—eight women and seven men (average age = 30 years, SD = 10.54)—five of them were high school graduate, nine had a bachelor's degree and one had a master's degree. All of them were employed in public or private companies. Three participants declared to be religious, while 11 were atheist and one agnostic. Moreover, seven of them saw death as annihilation, five of them as a passage toward another dimension, two were not sure and one expressed a more complex vision that integrated more than one aspect.

The recruitment criteria were being at least 18 years old and having watched during the lockdown period in Italy (from the 8th March to the 4th of May 2020) at least a movie or a TV series that addressed the theme of viruses, contagions, epidemics and their consequences. These could be either direct (movies in which the presence of un-dead people was explicitly referred to the effect of a virus – for example the movie “28 days later” by Danny Boyle) or symbolical (horror movies concerning zombies, that can be considered as a metaphor of contagion fears).

The movies or TV series participants actually watched were the following: *Contagion* by Steven Soderbergh (six viewers), *The Last Man on Earth* by Sidney Salkow (one viewer), *I am Legend* by Francis Lawrence (one viewer), *Resident Evil* by Paul W. S. Anderson (one viewer), *Outbreak* by Wolfgang Petersen (one viewer), *BirdBox* by Susanne Bier (one viewer), *Flu* by Kim Sung-su (one viewer), *Medici* (TV series) by Frank Spotnitz and Nicholas Meyer (one viewer), *The Walking Dead* (TV series) by Frank Darabont (one viewer), *The Hot Zone* (TV series), by James V. Hart (one viewer).

For more information concerning the movies watched by each participant and the exact period in which they did it, please see Table 1.

**Table 1:** Movie/TV series watched by participants.

**Title and typology**	**Pandemic period of viewing**	**Rewatched**
Contagion (realistic)	First month	Yes
Contagion (realistic)	Second month	No
Bird Box (fiction)	First Month	No
The Hot Zone (realistic)	First month	No
The Walking Dead (fiction)	Extended over time	Yes
Contagion (realistic)	First month	Yes
Medici (realistic)	Extended over time	No
Contagion (realistic)	First month	No
Contagion (realistic)	Second month	No
Flu (realistic)	First Month	No
The Last Man on Earth (fiction)	First month	Yes
Resident Evil (fiction)	First month	Yes
I am Legend (fiction)	First month	Yes
Outbreak (realistic)	First Month	Yes
Contagion (realistic)	Second month	No

Participants were recruited through social network (Facebook) via an announcement, which invited anyone who felt the need to watch films featuring epidemics to engage in the study. Subsequently, the first participants were asked to think whether they knew other people who had watched the same kinds of movies/TV series during that period, and, if that was the case, to invite them to take part in the study too, by contacting the researchers. In this way, an adequate number of participants could be reached thought what is typically called “snowball effect.”

The research followed the American Psychological Association's Ethical Principles of Psychologists and Code of Conduct and the principles of the Declaration of Helsinki, and it obtained the approval of the Ethics Committee for the research in Psychology of the University of Padua (Italy) (n. B2A86963B9F0C9D30F4B5A435F3C9570). Signed informed consent was obtained by each participant.

For the present study a qualitative approach was adopted (30), and more specifically, the principles of Interpretative Phenomenological Analysis (IPA) have been followed (31). In order to get as close as possible to the participants' perspective, IPA follows two processes: People are asked to give a meaning to their experiences through a detailed narration guided by the researcher; the researcher then tries to give a meaning to the sense itself that emerges from the participants' narrations (31, 32). Through this method, the researchers get as close as they can to the point of view of the person who experiences a certain phenomenon (33).

Participants took part in a semi-structured interview, aimed to explore the specific reasons that have led them to watch during the lockdown some movies or TV series that refer to the theme of epidemics, contagions, serious health risks caused by infectious diseases. The interview was structured in order to enable the researchers to investigate participants' precise cognitions and emotions during that peculiar period, as well as during the vision of the movie/TV series, and right after that. Moreover, the interview also explored the participants' perception and attitude toward COVID-19 and the related lockdown measures (their fears, anxieties, negative thoughts etc.), in order to evaluate whether they seemed to change after the vision of the movie/TV series.

In order to better understand the deeper meaning of the phenomenon, the researchers also investigated the participants' spiritual dimension, independently from the religiousness, and their personal representation of the idea of death (as total annihilation or as a passage toward another dimension) (34). The semi-structured format of the interview was used in order to allow a certain degree of flexibility for the researchers, who had therefore a track of the fundamental themes to explore but could at the same time allow participants to feel as free as possible to focus on the themes that were the most meaningful to them, without interfering too much with the spontaneous flow of the conversation (31).

Each interview lasted about 90 min and was conducted in Italian (participants' native language), through a phone call or by Skype or Zoom, in order to comply with the current lockdown and social distancing measures and to safeguard the participants' and the researchers' health.

The interviews were audio-recorded (with participants' permission), and subsequently transcribed verbatim, still in Italian, for the analysis. The written texts obtained in this way were then analyzed using thematic analysis, in order to examine them in terms of their fundamental contents (35). The texts were translated in English after this process, during the elaboration of the first draft of the present article by the authors, who were very careful to maintain the linguistic style and precise terms the participants had used in the original language (Italian).

The analysis proceeded through six main phases: engaging in preparatory organization; reading the texts deeply; coding data; interpreting themes; searching for alternative explanations; and producing the final report (36). The process was performed by highlighting some recurrent words or concepts that appeared to be particularly meaningful for a participant (since during the interview participants had repeated them often or had given emphasis to them), and by subsequently confronting these elements with the ones that could be found in the other participants' narrations, and subsequently grouping together into broader thematic categories the elements that appeared to be shared the most among the participants (37). The analysis was performed with the software Atlas.ti (38), which allows researchers to directly work on written texts, highlighting portions of them (which become “quotations,” that is, parts of the direct speech of each participant that are particularly meaningful) creating labels to insert in each text that can adequately represent its fundamental themes, and elaborating broader clusters of meaning comparing the data gained from each text. This analysis method allowed the researchers to also explore the possible relation between participants' age, gender or reported spirituality and attitude toward the idea of death and their psychological response to epidemic-related movies or tv series. In order to achieve this, the authors evaluated each main thematic category that emerged from the obtained analyzed data in terms of how many male vs. female, religious/spiritual vs. atheist participants had expressed thoughts and ideas that could be inserted into that peculiar category, comparing it with the characteristics of those who had highlighted different aspects of their experience and therefore could be considered linked to other thematic categories more.

The participants' age difference was also considered in the process. The Atlas.ti software allowed to compare fundamental elements of each thematic category and highlight shared or instead different aspects among them, such as, for each quotation related to a specific thematic category, the age, gender, and religious orientation of the participant who expressed it.

## Results

From the data analysis, four main issues emerged to describe the reason why participants watched epidemic-themed movies/TV series during the lockdown period: “Reducing the uncertainty,” “The not always successful attempt to exorcize the contagion,” “Identification and catharsis,” and “From fortuity to interest.” The numbers indicated in each quote in the following description of the results specify the interview number that corresponds to each participant (from 1 to 15) and the order number that refers to the quote on the working text in *Atlas.ti*. The reported names are fictitious to respect participants' anonymity. Quotations are partially camouflaged to impede any possible recognition of the participants' identity.

### First Main Reason: Reducing the Uncertainty

A first fundamental and deeply felt desire all participants reported was to reduce the extreme uncertainty that they felt during the epidemic spread and in particular during the lockdown period. Such a need could be somehow fulfilled by watching movies or TV series that represented, either directly or even only symbolically, the theme of viruses and scaring contagions.

This is precisely what happened to Leonardo, a man very passionate about cinema who lived the lockdown period in total isolation in order to protect as much as possible the people he loved. Living completely isolated for 2 months, he reported his desire to understand what was happening, and the fact that he could find a sort of explanation thanks to the movie: “I was really driven by the desire to understand. I mean, the movie followed a scientific approach, and when the films are able to explain scientifically a problem, they really allow you to understand what happens. In this case, I understood that COVID-19 is a tough fight and that there are some methods to respect if we want to win… Obviously it is not simple since we were not ready for this, however there are some people who are actually working in order to protect us from these problems, just like the movie perfectly describes. I feel better when I think of it” [1.36–1.54]. Paola expressed a similar need for information, as she narrated how her desire to watch a movie concerning epidemics was driven by the hope that the work of fiction could offer her the explanation she was looking for and not finding in real life: “I wanted to see this movie because I wanted to understand what might happen, so that I could understand something I did not know. You know, I was informed, and I had already read a lot about the coronavirus, but I thought that perhaps that movie could add something important” [15:25].

Paola had been informed of the virus spread since December 2019, due to her work. However, information had not been exhaustive, and she lived the subsequent lockdown with huge apprehension. Another meaningful experience has been reported by Daniele, a man who lived the lockdown with his origin family. He suffered from a huge anxiety because COVID-19 reminded him the health problem of his childhood: “Well, I have always considered ignorance to be the worst possible thing. I mean, you can die, but you can die either knowing what you are dying for or not, and I prefer to die knowing what got me and how I could have acted in order to avoid it” [6.37]. He also added: “We will probably worry more and feel more anxious, or more scared watching movies like *Contagion*. However, perhaps a person or two will also realize that what is represented in this movie is the exact copy of what is happening right now, maybe they will become more aware of the situation, perhaps having more faith in a future cure, in a solution, this is what I think” [6.38].

However, the possibility to receive useful information concerning the pandemic situation through the movies could also feed some negative emotions, especially because of the possibility to inevitably recognize some problematic aspects depicted in the work of fiction and also present in the reality, as Daniele reported: “Well, let's say it gave me a lot of anxiety. I mean, I'm astonished by the effect of a microscopical at a global level. You can catch a train, or a bus, or eat something strange, maybe you simply touch a pole in a bus to maintain your balance and just with that you could already have spread the virus to all the people on the bus. You can eat something and spread a mortal virus. All this is really similar to what films describes” [6.53]. Agreeing with the others, Rudy, a sick person who had to prepare himself for the self-isolation even before it was actually required by the government, said: “Watching these movies helps me to represent what can be useful to do in critical moments. They describe the difficulties but also the remedies to the problems” [14:28].

### Second Main Reason: The Not Always Successful Attempt to Exorcize the Contagion

A second kind of motivation reported by participants was that to diminish or somehow exorcize the growing anxiety they were experiencing during the lockdown, due to the constant news of high peaks of contagion and of countless deceased persons. Watching movies was a strategy to avoid panic. For example, Laura, who had recently returned from China, affirmed: “My boyfriend and I were getting ready to face the lockdown. I think that it is quite difficult to understand the dimension of the problem, so we watched the movies together in an ironic way. We tried to cope with this situation as if it was a film, that is, not too tragically” [10:19]. Similarly, Rosanna, a woman who was particularly scared of the obligation to go to her workplace, admitted that she chose that kind of movies “because they transform everything as a fantasy, and this reduces my anxiety. Really, during the day, I'm constantly scared because of my constant exposure to the risk of contagion in the workplace. In the evening I can imagine that everything is an improbable plot of a film” [4:24]. She also reported that the repeated vision of such films more and more times reassured her because doing so she could predict all the events and passages, till the end. Knowing first how everything would turn out gave her back in the evening all the sense of security she had lost during the day: “It is relaxing to know before everything, when a situation is particularly scaring. There is always a peaceful start, a moment of extreme crisis in which many people die, and everyone is in distress and worried for his/her loved ones. However, in the end the protagonists always survive, and this is very relaxing. So, it is like telling yourself a fairytale. It is possible to face this pandemic thinking that it is only a fairytale. And this soothes you anyway. I felt understood by the TV series, I felt someone was in my same situation, and they all survived” [4:46]. Rudy described the same exigence: “These movies reassure you, because there is the evil that scares you, however you already know that the goodness will win. And it is interesting to learn the strategies of goodness” [14:30].

However, not all the participants obtained such a calming and some of them actually reported an increase in the level of distress. Leonardo, for example, said: “Well it creeps you out too obviously, because it makes you think of what could possibly go wrong and become even worse in real life. It is distressing because you understand that the problem could be more difficult compared to that you imagine and so that it is possible you aren't able to solve it” [1:45]. Similarly, Daniele described his rage after the watching: “Well, the movies leave you with a bitter kind of feeling, because they make you aware of the fact that the government gives priority to the families, or to the scientists, or anyway those who are in charge and have enough power. These categories of people are evidently more lack of others. They describe only how life is easier for people who have some particular advantages” [6.25].

### Third Main Reason: Identification and Catharsis

The identification with the protagonists was the third main reason for watching scaring films, to see a part of their life and situation represented. Indeed, all the movies these participants chose to watch narrated episodes very similar to their experiences. For example, Veronica lived the first moments of the epidemic with great anxiety: “I totally recognized myself in the protagonist. In a very similar way, when something bad happens, I feel very distressed like the narration of this story. However, I try to be as rational as possible. Of course, it is very difficult and the fight between reason and emotions is excellently narrated by this movie. When I see scenes that describe this conflict and see that everything finishes well, I'm happy to be as I am” [3:28]. Similarly, Giorgio, who spent the lockdown in total isolation in his country house, explained that he chose to watch pandemic-related movies where protagonists had to face the crisis completely alone: “Well, one film in particular excellently described this condition, that is to be alone in facing the danger. There is this part where you can see how the protagonist relates to his dog, in a kind of egoistical way. He is completely alone so he needs the affection his dog can give him, but this makes his dog eventually run away. And I was feeling exactly like that, I kept looking for other people's support even though I could not visit them, and that was even a little unhealthy. I really felt the same sufferance the protagonist experiences, when something is happening outside your home, but you are blocked away from everything” [11:9]. Also Gianluca lived the lockdown in total isolation and wanted to re-watch a movie that represented the last man remained on Earth, since that perfectly mirrored how lonely he was feeling: “I thought I wanted to watch it again to see how it had aged and also to pretend to be inside the movie itself, I knew I could identify with it since the protagonist is alone, and I was there all lonely watching it in my living room. Somehow, seeing that someone was absolutely lonelier than me made me feel less lonely” [13:30]. Many participants explained that identification helped them have a catharsis, with the effect of an intense liberation. Veronica, for example, reported that this psychological dynamic allowed her to finally have the strength to accept the present condition and feel at peace with the situation of health emergency and uncertainty: “I had a fever and also hypertension during the lockdown, so I was terrified of being infected even though I never left my home. I was there trying to control my fears but on an emotional and psychosomatic level I was suffering a lot, until at a certain moment I freed myself saying ‘Ok, I feel really bad, denying this is pointless, let's embrace this situation of pain and stop’. Just like the protagonist did, that's why I really saw myself in her. Watching the movie has taught me that I am not the only one who has to live this situation and that in any case I do not miss anything to be able to handle it even if it is something absolutely unpredictable and unusual” [3:38]. Veronica also reported a peculiar feeling of hope she felt at the end of the movie, as if re-living all her suffering in the protagonist's shoes made her able to somehow detach from the situation and feel hopeful again: “The last scene, in which the protagonist tells her son that she is his mother and she says she is sorry for refusing to acknowledge that before, and you can see she is changed and wants to commit herself completely to her new, better life, that helped me too. After all the suffering, in the movie and in real life, that moment helped me see the situation in a slightly different way, in a more positive light. The suffering and the traumas permitted the protagonist to become a better person” [3:29].

However, in some cases the identification with the protagonist did not produce positive effects. Giorgio, for example, reported that he decided to watch a pandemic-themed movie because “that was the only way to feel some emotions since I could not speak to anyone. I needed to be able to feel something for situation like this. Indeed, I could identify myself with the protagonist and feel a stronger emotion” [11:10]. Instead, this experience increased the level of worry because he identified some behaviors depicted in the movie very similar to those in the real world and this threatened him: “I am rather convinced that…I mean, I was very scared. I felt a sort of hunt for those who were infected in the film and in parallel for those who suffer for the COVID infection around us. Now, when I read the news I cannot think that someone would exaggerate, and I am very scared that we have to live an experience very similar to that of the film. I think that we risk a sort of witch hunt so to speak” [11:24].

### Fourth Main Reason: From Fortuity to Interest

The last condition reported by some participants was perhaps the most peculiar one, since some of the people interviewed explained that they were not particularly interested in pandemic-related movies or TV series, which differentiates them from the majority of the other participants who actively looked for those movies. Simply they found a contagion-themed movie or TV series casually on television or on the Internet that caught their attention because of the pandemic period, so they decided to watch it. In some cases, the effects were really similar to those of participants who actively searched such a kind of films. In fact, one of the reasons was to be better informed and to understand what was happening, even without believing that they could have learnt something useful to manage the difficulties caused by COVID-19. Francesca said: “I do not deny that after watching the very first scenes I was intrigued. There were some moments that seemed exactly what we were living, so I got up widening my eyes and said ‘this is not the typical science fiction movie’, and it was obviously the whole context in real life that made me perceive it as a realistic kind of work” [15:25]. The same idea was expressed by Alessia, who was visiting her parents when the lockdown was announced so she was unexpectedly blocked at their home. She enjoyed the time with her family, but the impossibility to go outside and exercise made her accumulate a lot of tension and eventually gave her insomnia. She said: “I was simply dining, and I saw that this movie was beginning, and it starts with a damn cough, so that already caught my interest, and I watched it till the end. I found it unsettling, but also accurate, similar to our reality. Even though I am not really into that genre, I liked it because it made me think” [9:31–9:38]. Similarly, Annarita lived the lockdown period in a very distressful way with her origin family: “Those kinds of movies make me anxious, I would not usually watch them, however I saw that it was identical to what we were living, so I thought ‘Oh God, this is the same! That person seemed healthy and now is already dead, I need to see this’. It was very stressful, but I thought that I had to see it because it was a sign of what was happening and so I had to realize something” [2:32].

For these participants, feelings subsequent to the vision were mostly negative, as Annarita described:

“When you see it explicitly in a movie, see those common graves, you think ‘Oh God, I really could end like one of them during the first phase of the epidemics, the most acute one, or perhaps my father, thrown like an animal inside a common grave’. How can I change this situation? I do not know how to manage this tragedy” [2:51]. She also reported feelings of anger, because what she saw represented in the movie mirrored all the difficulties she found in real life during the pandemic: “Rage, so much rage, because people in the movie could not have access to face masks to protect themselves and that happened to me too. People who do not want to see those they know, just like happened to me. Being isolated by others. The human aspect of the movie made me suffer a lot, I suffered a lot because of the pandemic, and I see that people have become animals, or even, objects…I do not know, automatons. And I lived this firsthand. And I saw it also in the movie” [2:48]. However, at the end she admitted that: “Since the movie ends with the contagions decreasing, this gave me a little hope. I mean, if they could make it in the movie why can't we make it too? Perhaps not returning to our previous life, but at least being able to still have all the people I love alive and well six months into the future” [2:50]. Similarly, Alessia narrated: “The film reassured me because the effects of the virus were stronger in the movie than in real life. I mean, the effects on society, people who start to shoot each other to be able to eat. I felt reassured because I thought that it could have been worse in real life too, the virus could have been even more lethal, and we could have reached that terrible point showed in the movie” [9:16].

## Discussion

This study was aimed to explore the peculiar phenomenon of those people who, during the early phase of the COVID-19 pandemic in Italy, decided to watch a movie or TV series that represented situations of contagions, epidemics and health risks. This phenomenon, in fact, may seem to be incoherent to the general Western culture which removes any reflection on death, dying or human vulnerability (18, 32). However, as some researchers have highlighted, the typical balance people had reached between the vague awareness of the possibility of their death and the need to protect themselves from the anxiety this generated, was completely subverted by the unpredictability and devastating impact of COVID-19 pandemic. Instead, it constantly made death present and salient, forcing people to find new ways to cope with it (15, 22). Indeed, our results highlighted a peculiar way of dealing with the epidemic and its heavy consequences, since it was not possible anymore for the participants to simply ignore the thought of their mortality. In this way, cinema became (sometimes thanks to a personal passion as happened for Leonardo, sometimes even by pure coincidence, as happened for example to Annarita) a powerful tool that helped them face the lockdown period.

All participants shared the need to be more informed and to better understand what was happening. And while the medias and the governments seemed not to be able to offer proper explanations, pandemic-related movies assured some kind of insight, as reported by Leonardo, Paola and Daniele. As literature already discussed, films and movies permit to find a mirror that help people understand and interpret their current reality (25, 27). Another important motivation was for example reported by Laura, Rosanna and Rudy, who said they wanted to exorcize and therefore diminish their high levels of anxiety related to the current Italian lockdown situation. The possibility to detach from real life problems by seeing the situation in a different light, together with the identification with the protagonist, help to distance fear and anxiety, reaching a more peaceful psychological state (29). An unsettling reality becomes this way somehow easier to accept, thanks to this peculiar filter which cinema can put between spectators and their life, giving shape to those inner processes of thought that could result problematic before (39).

This element emerges also among those participants, like Veronica, Giorgio and Gianluca, who explicitly reported to have actively chosen a movie or a TV series in which there was a protagonist they knew they could identify with. This process, namely the need to identify with a fictional character and the consequent feeling of hope and faith in the future that came with it, caused a cathartic liberation, very similar to the one already described by ancient Greek philosophers and tragedians Plato, Aristotle, Aristophanes (40), and which has been already documented in scientific literature as a possible beneficial effect of cinema, especially in this current time of global pandemics (41, 42). Finally, there were also participants who, despite not having any initial intention to watch virus-related movie or TV series, found them by chance and eventually could not but being absorbed in because of the inevitable resemblance with their reality. These participants also reported, like the rest of the respondents, that watching those movies somehow fulfilled their need of being better informed and of gathering as much information as they could on the COVID-19 pandemic.

Watching movies or TV series epidemic-related during this peculiar historical period led also to experience some negative emotions, as reported by some participants. Daniele, Leonardo, Giorgio, Alessia and Annarita, for instance, described feelings of increasing anxiety and sometimes even rage, especially when they could identify some problematic aspects that were present in their real life too. This risk has already been highlighted by other studies (26). However, even those who experienced at first feelings of distress could eventually and most of the times report feelings of hope and relief by the end of the movies, since the already mentioned process of cathartic identification and the possibility to exorcize their anxiety could counterbalance their initial increase in fear and negative emotions (for a more precise summary of most participants' feelings after the movies/tv series, please refer to Table 2 at the end of the present section).

**Table 2:** Feelings and emotions after the vision.

**Participant**	**Gender**	**Age**	**Movie/Tv series watched (typology)**	**Consequent emotions/ feelings**	**Quotations**
Rosanna	Female	54	The Hot Zone (Realistic)	Comforted, feeling understood	“It is relaxing to know before everything, when a situation is particularly scaring. […] in the end the protagonists always survive, and this is very relaxing. So, it is like telling yourself a fairytale. It is possible to face this pandemic thinking that it is only a fairytale. And this soothes you anyway. I felt understood by the TV series, I felt someone was in my same situation, and they all survived”
Rudy	Male	49	Outbreak (Realistic)	Reassured	“These movies reassure you, because there is the evil that scares you, however you already know that the goodness will win. And it is interesting to learn the strategies of goodness”
Leonardo	Male	45	Contagion (realistic)	Distressed	“Well it creeps you out too obviously, because it makes you think of what could possibly go wrong and become even worse in real life. It is distressing because you understand that the problem could be more difficult compared to that you imagine and so that it is possible you aren't able to solve it”
Daniele	Male	23	Contagion (realistic)	Upset, anxiety	“Well, the movies leave you with a bitter kind of feeling […] it gave me a lot of anxiety. […] You can catch a train, or a bus, or eat something strange, maybe you simply touch a pole in a bus to maintain your balance and just with that you could already have spread the virus to all the people on the bus. You can eat something and spread a mortal virus”
Gianluca	Male	22	I am Legend (fiction)	Feeling less lonely	“I thought I wanted to watch it again to see how it had aged and also to pretend to be inside the movie itself, I knew I could identify with it since the protagonist is alone, and I was there all lonely watching it in my living room. Somehow, seeing that someone was absolutely lonelier than me made me feel less lonely”
Veronica	Female	25	Bird Box (fiction)	Hope, seeing the situation in a more positive light	“The last scene, in which the protagonist tells her son that she is his mother and she says she is sorry for refusing to acknowledge that before, and you can see she is changed and wants to commit herself completely to her new, better life, that helped me too. After all the suffering, in the movie and in real life, that moment helped me see the situation in a slightly different way, in a more positive light. The suffering and the traumas permitted the protagonist to become a better person”
Giorgio	Male	24	The Last Man on Earth (fiction)	Apprehension, fear	“[…] I was very scared. I felt a sort of hunt for those who were infected in the film and in parallel for those who suffer for the COVID infection around us […] I think that we risk a sort of witch hunt so to speak”
Annarita	Female	26	Contagion (realistic)	Evolution, from anxiety and rage, to final hope at the end of the movie	“When you see it explicitly in a movie, see those common graves, you think ‘Oh God, I really could end like one of them during the first phase of the epidemics, the most acute one, or perhaps my father, thrown like an animal inside a common grave’ […].” “Rage, so much rage, because people in the movie could not have access to face masks to protect themselves and that happened to me too. People who do not want to see those they know, just like happened to me.” “Since the movie ends with the contagions decreasing, this gave me a little hope. I mean, if they could make it in the movie why can't we make it too? Perhaps not returning to our previous life, but at least being able to still have all the people I love alive and well six months into the future”
Alessia	Female	24	Contagion (realistic)	Reassured	“The film reassured me because the effects of the virus were stronger in the movie than in real life. […] I felt reassured because I thought that it could have been worse in real life too, the virus could have been even more lethal, and we could have reached that terrible point showed in the movie”
Laura	Female	25	Flu (realistic)	Possibility to perceive the situation in an ironic way	“My boyfriend and I were getting ready to face the lockdown. I think that it is quite difficult to understand the dimension of the problem, so we watched the movies together in an ironic way. We tried to cope with this situation as if it was a film, that is, not too tragically”

Lastly, no noteworthy difference was detected among participants based on their age, gender or reported spirituality and attitude toward the idea of death, in relation to their response to epidemic-related movies or tv series and the psychological effects they had on them. Even though the number of participants is small, which does not permit a generalization of the results, this element could however suggest that the cathartic power of cinema, especially in moments of peculiar crisis as the present COVID-19 pandemic represents, can be equally beneficial for different populations, regardless of other personal characteristics.

## Conclusions

The current COVID-19 pandemic has had a devastating impact on people's physical and psychological well-being, with an increasing mortality salience that challenges Western Societies' tendency to occult death and dying. In this situation cinemas become powerful tools capable to manage the anxiety and the anguish caused by the epidemic and the consequent restrictive measures. The identification and projective processes enacted by movies allow people to partially detach from their own sorrows and enter a fictitious dimension that may at the same time offer them useful insights into the pandemic and free them from their negative emotions. Cinema appears then to be a useful instrument to help people cope with pandemics.

However, it has to be underlined that our study has some limitations, in particular the small number of participants, typical of a qualitative study, does not allow to explore completely the possible different influence of variables such as participants' age, gender or spirituality in relation to the psychological effects of cinema.

In the near future, therefore, it would be interesting to conduct similar interviews again, thus expanding the number of participants, in order to possibly include more peculiar points of view and explore more in depth these aspects.

## Data Availability Statement

The raw data supporting the conclusions of this article will be made available by the authors, without undue reservation.

## Ethics Statement

The studies involving human participants were reviewed and approved by The Ethical Committee for Psychological Research of the University of Padua. The patients/participants provided their written informed consent to participate in this study. Written informed consent was obtained from the individual(s) for the publication of any potentially identifiable images or data included in this article.

## Author Contributions

IT: project ideation, research design, supervision, analysis, article writing, and coordination. ER: project ideation, interviews, analysis, and article writing. SP: analysis, supervision, and article writing. IM: supervision and consultation during the second revision of the manuscript. HO: supervision and article writing. All authors contributed to the article and approved the submitted version.

## Conflict of Interest

The authors declare that the research was conducted in the absence of any commercial or financial relationships that could be construed as a potential conflict of interest.
